# Assessment of the Impact on Human Health of the Presence of Norovirus in Bivalve Molluscs: What Data Do We Miss?

**DOI:** 10.3390/foods10102444

**Published:** 2021-10-14

**Authors:** Federica Savini, Federica Giacometti, Federico Tomasello, Marta Pollesel, Silvia Piva, Andrea Serraino, Alessandra De Cesare

**Affiliations:** Department of Veterinary Medical Sciences, University of Bologna, Via Tolara di Sopra 50, 40064 Ozzano Emilia, Italy; federica.savini3@unibo.it (F.S.); federico.tomasello4@unibo.it (F.T.); marta.pollesel@studio.unibo.it (M.P.); silvia.piva@unibo.it (S.P.); andrea.serraino@unibo.it (A.S.); alessandra.decesare@unibo.it (A.D.C.)

**Keywords:** Norovirus, bivalve mollusc, risk assessment, food-borne virus, food microbiology, water-borne virus, microbiological criteria

## Abstract

In the latest One Health ECDC EFSA technical report, Norovirus in fish and fishery products have been listed as the agent/food pair causing the highest number of strong-evidence outbreaks in the EU in 2019. This review aims to identify data gaps that must be filled in order to increase knowledge on Norovirus in bivalve molluscs, perform a risk assessment and rank the key mitigation strategies for this biological hazard, which is relevant to public health. Virologic determinations are not included in any of the food safety and process hygiene microbiologic criteria reflected in the current European regulations. In addition, the Escherichia coli-based indices of acceptable faecal contamination for primary production, as well as the food safety criteria, do not appear sufficient to indicate the extent of Norovirus contamination. The qualitative risk assessment data collected in this review suggests that bivalve molluscs present a high risk to human health for Norovirus only when consumed raw or when insufficiently cooked. On the contrary, the risk can be considered negligible when they are cooked at a high temperature, while information is still scarce for non-thermal treatments.

## 1. Introduction

In 2019 Norovirus (NoV) was associated (with other Caliciviruses) with 457 outbreaks and in 22.5% of total cases with related illnesses, accounting for one in five of all outbreak-related illnesses in the EU [1]. In the same year, outbreaks caused by NoV increased by 13.1% in respect to 2018. Indeed, it was identified as the second most frequently reported causative agent in food borne outbreaks in Europe after *Salmonella* spp. [1] NoV in fish and fishery products have been the agent/food pair causing the highest number of strong-evidence outbreaks in EU in 2019 [1]. Worldwide, Noroviruses are the most common etiologic agent of acute gastroenteritis, causing an estimated 685 million illnesses [2].

Virus transmission can occur either from person to person via the faecal–oral route, or via contaminated food, surfaces and water. Among the variety of foods at risk, usually due to contact with fecally contaminated water bivalve molluscs, ready-to-eat leaf vegetables, soft fruits and fresh produce are most commonly associated with foodborne outbreaks [3]. Indeed, not only in secondary-treated municipal wastewater of up to 4 log titer of human NoV GI and GII particles per mL have been described [4], but also treatments with UV or membrane filtration (the so-called tertiary treatment) have been shown not to reduce NoV surrogates to a non-infectious level [5]. Bivalve molluscs feed by filtering large amounts of water through their gills. This causes the accumulation of pathogens to levels considerably higher than those in the overlying waters [6]. 

In Europe, in relation to the sanitary control of shellfish produced and sold for human consumption, areas in which bivalve molluscs are cultivated in marine or brackish water are defined only on the basis of bacterial indicators, predominantly centering around the sanitary classification of harvesting areas into the three categories—A, B or C—based on increasing *Escherichia coli* concentration, that are routinely used to test for microbiological quality (Reg. EC 625/2017; 627/2019). For each classification category, different degrees of post-harvest action are required: from no additional treatment for shellfish harvested from class A waters, whereas class B and C shellfish require a process of depuration, relaying, as well as thermal or non-thermal treatment prior to sale.

In relation to food safety criteria concerning bivalve molluscs placed on the market, the non-detection in 25 g for *Salmonella* and the detection of *E. coli* between 230 and 700 MPN in 100 g flesh and intravalvular liquid are mandatory (Reg. EC 2073/2005 and 2285/2015). Nevertheless, *E. coli* levels may not be correlated with the presence of viruses [7,8,9,10,11,12,13]. For some authors, *E. coli* provides a useful indication of the likelihood of contamination with NoV [14], while others state that *E. coli* levels may not be correlated with their presence [10,15,16,17,18,19,20]. In any case, class A status is not a guarantee of the absence of NoV contamination.

Since 2010 the quantitative real-time PCR- ISO (15216-1:2017) method has been introduced to detect and quantify NoV (and HAV) from foodstuffs or food surfaces. This standard allows a fast detection of virus genomes with low false positive rates. Nevertheless, a lack of correlation between the presence of RNA and infectivity is reported [21], since RT-qPCR detection is focused on the viral genome and does not give information concerning the structural integrity and infectivity of the overall particle [22,23,24]. Furthermore, a quantitative PCR does not allow us to constantly identify the relationship between the number of infectious virus particles and the number of virus genome copies detected, and thus the infectious risk associated with low level positive oysters, as determined by real-time PCR, may be overestimated [25]. Currently, no threshold infectivity limit is established for NoV detected by PCR. Moreover, virologic determinations are not included in any of the food safety and process hygiene microbiologic criteria reflected in European regulations; thus, a profound concern on the subject has been expressed from EFSA in recent documents related to the occurrence and control strategies on foodborne viruses [13,26,27].

A major threat is posed by the hypothesis that the burden of NoV is likely to increase as a consequence of climate change and population growth, since not only pathogen load, but also its survival in the environment, is associated with increasing rainfall/runoff [28]. Indeed, outbreaks of waterborne infectious diseases do occur following extreme water-related weather events in both developed and developing countries [29], mainly due to an overloading of the sewer network: when rainwater drains into the sewers, it carries domestic sewage and industrial wastewater. Consequently, untreated sewage flows into rivers, lakes or coastal areas [30]. Several studies have demonstrated that untreated wastewater, flooding and runoff during high precipitation events are a source of faecal contamination and seriously impact the shellfish area [28,31,32,33,34,35,36,37]. Persistence of poor shellfish quality for several weeks, namely viral contamination, has been described as occurring after a winter rainfall event [38]. Different factors have been suggested to influence virus survival in the water column: hardness, solar radiation and phosphate levels are the major ones [39,40,41,42]. Above all, temperature and UV irradiation are the environmental parameters that most affect viral particle stability in seawater, while salinity is considered to be a secondary factor in viral inactivation [43,44].

NoV genome persistence shows a temperature dependency [45,46,47,48], with a general better persistence at cold temperatures (4 °C) in both drinking and wastewater [49], but also the nature of the matrix seems to play a significant role [49]. A faster decay in wastewater is attributable probably to the higher presence of organic matter and indigenous microorganisms than in drinking water [50,51]. NoVs survival has been documented to be up to 2 months in groundwater supplies [52] and is still infectious after this time [53]. Very little difference in survival has been demonstrated under sunlight or dark conditions at temperatures ranging from 9 °C to 11 °C, but a more rapid NoV inactivation under sunlight rather than in the dark has been evidenced, with temperatures reaching 16 °C to 18 °C [8]. Indeed, during the winter season, a typical NoV peak is registered in sewage [54]; this might be correlated with the higher incidence of human outbreaks [52], but also with the colder temperature of the water and a lower UV irradiation.

To date, human-to-animal transmission has been reported only in dogs [55,56], but a broad range of mammalian and bird species can be susceptible to human NoV [57]. On the contrary, no report of human infections of animal noroviruses is known, but some serological studies have reported sero-prevalence against bovine [58,59,60] and canine [61,62] NoV in humans. Hence, the zoonotic potential of NoV transmission, mainly from animals farmed for human consumption, but more in general from mammals to humans via the food chain, cannot be neglected [63]. Most importantly, bivalve molluscs are identified as “hotspots” for the accumulation of multiple NoV strains [64,65], presenting opportunities for human co-infection, with consequent recombination of viral strains, thus being high-risk reservoirs of novel recombinant strains into the human population [66,67]. As an example, GII.4 NoV strains continuously undergo genetic/antigenic diversification, periodically generating novel strains through accumulation of punctuate mutation or recombination [68].

The assessment question being addressed in this review was formulated as: “Do bivalve molluscs present on the market represent a NoV infection risk for the consumers?”. In line with the objective to identify the data gaps in performing a full risk assessment, the information has been collected following the key steps as outlined by the Codex Alimentarius Commission.

## 2. Hazard Identification

### 2.1. Etiologic Agent—The Food/Hazard Combination Addressed by This Review Is “Human Norovirus in Bivalve Molluscs”

The genus Norovirus (Fam. *Caliciviridae*) comprises genetically diverse viruses infecting a wide range of mammalian host species [69,70,71,72,73,74,75,76]. NoVs are grouped based on the major capsid protein VP1 into 10 genogroups (GI-GX), and are further divided into 48 genotypes [77]. Among them, only GI, GII and GIV are known to infect humans, with genogroups I and II having the greatest epidemiological impact [78,79,80]. The phylum Mollusca is regarded as the second most prevalent animal phylum, with eight classes comprising bivalves (oysters, scallops, mussels, and clams), cephalopods (octopus, squid, and cuttlefish), and gastropods (whelks, sea snail, abalone, and cockle) that represent the economically significant molluscs [81]. Within gastropods, raw sea snail consumption, compared with bivalves, have been considered to present little risk of NoV infection [82], while in squid, cuttlefish and octopus, the highest-risk parts are removed and tend to be cooked before being consumed, reducing or eliminating any Norovirus contamination that may be present [83].

Based on EU Reg 853/2004, live bivalve molluscs means filter-feeding lamellibranch molluscs. The species of molluscs considered were only those that act as bio accumulator, since they can concentrate different types of pathogens in their tissues due to their filtering capacity [84,85]. These include clams, mussels and oysters. Among them, specific binding to carbohydrate ligands have been demonstrated for oysters [33,86,87], which allows them to concentrate viruses up to 99 times compared with the surrounding water [6] within digestive and non-digestive tissue cells [88]. Specifically, NoV GI is concentrated in a more active and efficient way than GII strains. Higher loads of NoV GI are usually measured compared to NoV GII in both oyster and mussel samples [12,25], while in shellfish-borne outbreaks, a relatively higher frequency of GI strains is described [86]. This suggests that bivalve molluscs represent a more potent reservoir for the transmission of norovirus GI compared to GII [87] as the result of a different affinity to mussel tissues that may influence their ability to bioaccumulate [89,90]. A higher prevalence of GII than for GI has been described in the periods January to February and November to December, while the concentration was lower between July–August and September–October [25]. The higher apparent prevalence of GII described during winter may be a reflection of the fact that a higher prevalence of this genogroup is also described in the human population in the same period [91]. In addition, during summer, the apparent prevalence of GI may remain more constant due to a slower clearance of these viruses given their specific binding to oyster tissues [92].

### 2.2. Prevalence—GI and GII, Circulating either Simultaneously or Separately, Are the Only Genogroups Detected in Bivalves

HuNoVs prevalence in bivalve molluscs worldwide has been reported to lie within the range of 0–95.6% [93,94]. Such a huge range might be also attributed to the application of different laboratory protocols that use different extraction methods or different primers [95]. Data obtained from ready-for-consumption bivalve molluscs (i.e., sold in markets) report very different levels of contamination that range from 2.3% [96], 3,4% [97] to 23.10% [98], but also 54% [99].

Usually, multiple different viruses can be found co-circulating, but large epidemics and spreads to different countries are mainly caused by a single virus [100]. In this regard, since the mid 90s, GII4 has become the predominant NoV genotype, and some of its variants have spread globally [91,101]. The pandemic variant GII.4 Sidney 2012 has been circulating since its emergence in 2012 [68,102,103], while during 2014 until winter 2015, a novel NoV GII.17 variant, after a sudden emergence, became prevalent not only in Eastern Asia [104], but also in other regions [105]. Indeed, the GII.17 variant (strain Kawasaki 2014) has subsequently been reported in several countries, including Australia, Hong Kong, Taiwan, North America, France, Italy, the Netherlands, Japan, New Zealand, and Russia [106,107,108,109,110,111].

It is important to underline that data concerning viral strains may be affected by their concentration, namely, if more than one type is present in a sample—as frequently outlined in food or environmental samples—the assay successfully amplifies the type that is either more concentrated or towards which the primers show a higher affinity [112]. In this regard, an important step would be to perform a targeted epidemiological investigation in order to anticipate the emergence of novel variants in preparedness for upcoming epidemics.

Available data concerning concentrations of ready-for-sale oysters and mussels demonstrate that the Log10 mean NoV genome copy numbers are comprised within the same order of magnitude in different European countries such as the Netherlands, UK and Italy [12,25,113]. A comprehensive analysis conducted in Italy on mussels, clams, oysters and other species, revealed that the average contamination level ranged from 3 × 10^0^–3.0 × 10^3^ copies/g in samples from class A areas, while from 3.3 × 10^1^ to 1.5 × 10^4^ copies/g samples from class B areas [113]. In Spain, Polo and collaborators performed quantification on different species: wild mussels showed the highest average values (6.5 × 10^3^ RNAc/g) followed by cultured mussels (4.2 × 10^3^ RNAc/g), clams (3.5 × 10^3^ RNAc/g) and cockles (2.1 × 10^3^ RNAc/g) [114]. The higher accumulation performance has also been described by Suffredini and collaborators [115]. In China, in shellfish at retail (species included *Crassostrea gigas*, *Mytilus edulis*, *Azumapecten farreri*, *Sinonovacula constricta*, *Scapharca subcrenata*, and *Ruditapes philippinarum*), the quantity range was between 1.9 copies/g shellfish meat to 7.94 × 10^5^ copies/g shellfish meat [116]. Nevertheless, a study comparison is affected by the absence of standardization, namely, a geometric or a mathematic mean is arbitrarily applied, as well as the use of log transformation.

### 2.3. Pathogenesis

Given the highly contagious nature of hunovs, the identification of the virus and its source must be immediately identified once an outbreak starts, in order to control the damage [78]. 

Ingestion of food contaminated by NoV causes an infection, where a sero-response is mounted after colonization of intestinal tissues [117], but a subpopulation of individuals is resistant to infection and disease. Indeed, NoVs were the first viruses showing different infection risks depending on host genetics [118], being largely dependent on the presence or absence of human histo-blood group antigens (HBGAs) on gut epithelial surfaces. As a consequence, pathogenesis can be divided into two distinct conditions: secretor negative (Se−) individuals: infected without presenting any symptoms, with a small infection risk at high doses, especially for GI viruses. Asymptomatic shedders; secretor positive (Se+) individuals: after infection, (in secretor-positive subjects, GI viruses appear slightly more infectious than GII viruses) individuals typically become symptomatic after 24–48 h: acute vomiting, diarrhea, and abdominal cramps [119], while only 40% of NoV cases report fever [120,121]. Illness usually resolves after 48–72 h [117] but elimination of the virus can persist for weeks or months after recovering. Peak levels and duration of shedding have shown considerable individual variation with no difference between symptomatic and asymptomatic infections [122]. Median NoV GII viral loads in the range of 1.14 × 10^7^ to 3.81 × 10^8^ copies/g stool have been evidence in patients with acute gastroenteritis [123,124,125], but loads up to 10^9^ genomic copies/g [126] in faeces of both infected symptomatic and asymptomatic patients might be excreted [127], thus contributing to the virus dispersion in the environment.

### 2.4. Exposure Pathways—Bivalve Shellfish Are Harvested within Estuaries and Coastal Zones 

The only known reservoir for human norovirus is human faeces that can contaminate coastal environment through discharges from municipal sewage treatment plants, on-site sewage systems or urban runoff. HuNoV concentrations in raw wastewater as high as 3.4 × 10^9^ genome copies/L have been measured [128]. Currently, sewage treatment such as chlorination or ultraviolet irradiation (UV) may not be designed for effectively removing viruses such as NoV [5]. Indeed, UV treatment can lead to a ~2 log10 reduction [129], but removal efficiency is dependent on viral load [28,130]. The most important factor affecting the reliability of this disinfection method is the efficiency of upstream processes, the application of a suitable wavelength and dose for a sufficient period of time [37]. Regarding chlorination, discrepancies exist in the literature [131], and this might be due to the protective action of water turbidity [132,133] or water quality [134], but preparation of the virus in benchmark studies may have also interfered with the obtained results [135]. In addition, after treatment, even though the viruses are inactivated, particles can remain in sewage effluent and can be detected by genetic analysis.

Importantly, dissimilar virus dispersion and presence in the environment are described, and this may be due to the unequal distribution of NoV gastroenteritis in Europe [136,137], but geographical factors such as distance from the coast, from rivers [138], seawater temperature, salinity, and land runoff may also play a role in different contamination levels of the harvesting [139].

NoV illnesses related to shellfish consumption generally show a peak incidence during the wintertime, presenting a seasonal pattern [140,141]. During non-epidemic periods, less than 10^3^–10^4^ genomic copies/liter of NoV are present in treated wastewaters, while during winter the concentration is probably 100- to 1000-fold higher [142,143,144]. In an analogy with the seasonal trend, NoV levels typically peak in winter in sewage [54,145], freshwater [146,147], and seawater [54,148]. Indeed, colder water temperatures, increasing stability of viruses, and reducing exposure to solar irradiation [52] facilitate NoV persistence in waters.

The risk of the presence of infectious Norovirus in marketed bivalve molluscs has been assessed considering pathways that might interfere both with virus concentration and viability.

Molluscs collected from B and C areas might be thermally processed or subjected to relaying to meet microbiological criteria consistent with food safety criteria before being placed on the market as live animals. The relaying and depuration processes are commercially important given the habits of the consumers, who prefer to eat oysters live/raw and clams and mussels lightly cooked [149,150,151]. In any case, based on chapter V of Reg. 853/2004 on health standards for live bivalve molluscs, food business operators must ensure that live bivalve molluscs placed on the market for human consumption meet the standards of organoleptic characteristics associated with freshness and viability, including shells free of dirt, and an adequate response to percussion and normal amounts of intravalvular liquid.

Considered exposure pathways are described below:

#### 2.4.1. Pathway 1—Live Bivalve Molluscs That Did Not Undergo Any Treatment after Being Harvested (Only for Class a Harvesting Area)

Placing on the market as live bivalve molluscs may only occur without post-harvest treatment, for those harvested from a class A area. Norovirus is normally present in shellfish harvested in a class A area. Studies report analysis performed among oysters, mussels and clams with a 10.5% of positivity [138]. For oysters, the apparent prevalence was lower in samples collected from class A production areas than in samples collected from other classes in all sampling periods; however, class A areas are not a guarantee of the absence of NoV contamination [25].

#### 2.4.2. Pathway 2—Live Bivalve Molluscs after the Depuration Process

Depuration is performed only for bivalves collected from class B areas. The process entails exposing the shellfish to clean seawater, commonly treated with chlorine, ultraviolet light or ozone, performing a rapid and effective reduction of the levels of *E. coli*. However, despite the achievement of bacterial end-product standards, depuration may not be effective in safeguarding against viral contamination [13,152,153,154]. In the European Union, depuration requirements vary according to the classification of harvesting areas but minimum time and water temperature are not stipulated for commercial depuration [155], which lasts generally 24–48 h [156]. Viral depuration is usually considered to be be ‘two phase’, where elimination in the first few days is more rapid than in subsequent days [157,158,159,160]. The first rapid phase is likely related to physiological traits related, including the filtration and clearance rate of the species, the digestion rate, and the enzymatic activity to the shellfish species involved, which are common to both bacterial and viral depuration [156]. Importantly, different parameters for each shellfish species influence—mainly the “first”—filtration efficiency such as temperature, salinity, dissolved oxygen content and turbidity [161]. In this regard, not only different optimal temperatures are specific for each shellfish species [162], but also the genetic makeup and geographical location will determine the range of temperature in which pumping will occur. More in general, the seawater temperature for depuration should not vary by more than 20 °C from that of the seawater harvesting area. Parallel to this, salinity of the seawater used for depuration should not vary by more than 20% from that of the water where the shellfish were harvested [161], but different genotypes of mussels may affect filtration rates [163]. Different behavioral responses to hypoxia have been documented between different species [164,165,166]; even though the oxygen concentration range for depuration is wide, oxygen levels should not fall below 2 mg/L [161].

In mussels, contrasting results have been obtained from studies conducted with similar depuration and concentration parameters. Indeed, the successful removal of a NoV GII surrogate was evidenced after 7 days [167], while human-derived Norovirus GII concentrations remained similar to the ones at the start of depuration after 4 days [11], in *M. galloprovincialis* and *M. edulis* respectively. Again, in another study, no significant differences were observed between depurated and non-depurated samples of *M. galloprovincialis* harvested in Italy [97]. Furthermore, an analysis at different points in the food chain demonstrated the stable presence of NoV GII in ready-for-sale bivalve molluscs over time (from 2013 to 2017) in samples of *M. edulis* taken in the Netherlands [12].

In the case of clams, different analyses have been performed on different species, but depuration has never eliminated viral contamination. Experimentally designed depuration after contamination of *Chamelae gallina* with Murine Norovirus (MNV) demonstrated the inability of the circuit to reduce the viral level after 72 h [168]. Similar results were obtained with samples of *T. decussatus*, which showed no statistically significant differences between depurated and non-depurated ones [97]. A trial conducted on Manila clam (*Venerupis philippinarum*) samples showed a percentage of significant removal between 24 and 72 h, but the remaining virus was still infectious at the end of the process [167]. In addition, NoV contamination was detected in all samples of *C. gallina* and *T. philippinarum* from class B, without significant differences between the seasons [169]. Studies on in-tank depuration of NoV (GI and GII) in oysters report very different results: from the reductions to negligible values within periods of 23 h, 10 and 14 days, to the persistence after 24 h [170] up to 29 days of continuing depuration [171,172]. A meta-analysis of published depuration experiments also suggests that the process requires more than nine days to achieve a tenfold reduction in NoV and HAV load [10]. Moreover, salinity, temperature and viral genogroup can highly influence depuration times in oysters [173].

#### 2.4.3. Pathway 3—Presence of Norovirus Particles in Live Bivalve Molluscs after a Relaying Process

Relaying involves moving live shellfish from their growing area to an area with cleaner waters, namely any sea, estuarine or lagoon area with boundaries clearly marked and indicated by buoys or any fixed means and used exclusively for the natural purification of live bivalve molluscs. Reg. EU No 853/2004 states that “food business operators must immerse live bivalve molluscs in seawater at the relaying area for an appropriate period, fixed depending on the water temperature, which period must be of at least two months’ duration unless the competent authority agrees to a shorter period on the basis of the food business operator’s risk analysis”. The WHO [174] suggests a relaying period of two months. The literature reports that a successful reduction in NoV contamination levels is achieved over a 17 days period in areas with clean seawater, but longer periods of 3 to 4 weeks have also been suggested [10]. Indeed, the time required depends not only on water temperature, but also on the extent of contamination and the shellfish species [154].

#### 2.4.4. Pathway 4—Presence of Norovirus Particles in Bivalve Molluscs after Thermal Treatment

Heat treatments ensuring that pathogenic microorganisms are eliminated can be industrially applied by shellfish producers to fulfill current EU regulations for samples collected from class B and C production areas. It has been suggested that thermal treatment are associated with changes in the virus capsid [175].

Since cell-culture systems to propagate HNoV in laboratories are not easily available, most of research on NoV stability after thermal treatment relays on viral surrogates, such as Tulane virus (TV), Feline Calicivirus (FCV) and Murine Norovirus (MNV). Contrasting results have been obtained regarding D-values [176,177,178], suggesting that not only do the different viruses act differently in the same food matrix, but also that the latter might have a protective role in some cases [175], as was speculated for oysters [177]. Even though they are scarce, available data on Z-value calculated among bivalve molluscs are mostly in accordance [177,179,180].

Criteria for bivalve molluscs require raising internal meat temperatures to a minimum temperature of 90 °C, to be maintained for a minimum of 90 s. Among commercial processes, even though they are compliant with the above requirement, the rates of temperature increase during heating and decreases during cooling (i.e., before and after the period of 90 °C for 90 s) can vary, leading to significantly different virus inactivation, which does not assure a common specified level of consumer protection [181]. Although the criteria may deliver variable degrees of virus inactivation, there are no reported human outbreaks of infectious illness associated with bivalve molluscs commercially processed accordingly to the legislative requirements [27].

#### 2.4.5. Pathway 5—Presence of Norovirus Particles in Bivalve Molluscs after Non-Thermal Treatment

The main aim of non-thermal processing is to assure food safety. The popularity of these methods in the food industry has increased following consumer demands for minimally processed foods and to circumvent the negative effect of heat on the nutritional properties of foods [182].

High pressure processing (HPP) is a non-thermal intervention for prominent food-borne pathogens associated with raw bivalve shellfish [183,184]. Currently, the pressure used to treat commercial shellfish is 275–300-MPa applied for several minutes, but oysters still taste good when treated up to 400 MPa [182,183]. Indeed, in general, a lower pressure extends shelf life, while high pressure conditions can change the appearance and texture of a product by pumping water in and making the meat appear bigger and softening its texture [184].

Regarding the human NoV surrogates MNV-1, FCV and TV an HPP treatment at 400 MPa, 4 °C for 2 min, can be effective in inactivating them in aqueous medium and/or oysters [185,186,187]. In addition, the exposure of clams to 500 MPa for 1 min at 20 °C did not alter the visual impact of the clam and the consistency of the flesh but led to obtaining a MNV free product [188]. On the contrary, a study performed with laboratory contaminated oysters on human volunteers demonstrated that HNoV has s higher stability since prevention of the infection could only be obtained in high pressure conditions (600 MPa, 6 °C, 5 min), in contrast with the lowest (400 MPa, 6 or 25 °C, 5 min) where, even though it was in a reduced number, volunteers became sick [189]. These results are in line with the finding that human NoV may be more pressure stable than its surrogates [190]. In general, the effectiveness of HPP for the inactivation of foodborne viruses depends on factors related to virus type; HPP parameters (pressure, temperature, or holding time); and food characteristics (food composition, pH and water activity of foods). Enveloped viruses are less resistant than non-enveloped viruses [190], and the differences are reported among NoV genogroups, with GI.1 strain presenting a higher resistance to pressure than GII.4 [191,192]. Regarding HPP parameters, increasing either the pressure or temperature of the process can enhance the inactivation of viruses. Temperature can act either synergistically or antagonistically with pressure for the inactivation of specific target viruses [186,191,193]. Refrigeration temperatures can enhance inactivation also by several logs [186]. Specifically, the pressures usually applied in commercial plants (300 MPa or below) in oysters experimentally inoculated did not result in substantial inactivation of GI.1 and GII.4 HuNoV if applied at room temperature but if lower (refrigeration) resulted in being effective [194]. On the contrary, a 400 MPa pressure applied for 5 min at 25 °C caused a 1.87 log_10_ and 1.99 log_10_ reduction of NoV GII.17 loads. An increase in HPP pressure levels can result in a higher inactivation of viruses as compared to those of an increase in time [195]. Different pressure values [193,194] have been applied for delivering a virus-free product in similar works, generating different results. Nevertheless, differences among the studies, such as the species of shellfish/the material tested, the equipment used for the hyperbaric treatment and the procedure used for the identification of the infecting virus may have contributed to the generation of different discrepancies.

For Human Norovirus, low pH levels appear to reduce HPP inactivation [196] but contrasting results have been obtained at different pH values for NoV and surrogates [191,197]. Indeed, the mechanism of the pH effect on the HPP pressure inactivation of various non-enveloped viruses is unknown and may be dependent on the capsid protein structure of the viruses [187]. Importantly for bivalve molluscs, survival of viruses is generally higher in foods with high activity water (a_w_) [198] and salt may play a protective role [199], maybe due to the stabilizing effect of NaCl on the capsid proteins of viruses during the HPP treatment [200]. Importantly, since inactivation is mainly achieved thorough changes in the function of capsid binding proteins, RNA integrity is not affected [185,197], posing a diagnostic challenge regarding the viability of the detected virus.

Reduction of NoV in fresh oysters has also been achieved following non-thermal dielectric barrier discharge plasma treatment for 30 and 60 min without a change in quality, as assessed by pH and Hunter colors [201].

## 3. Exposure Assessment 

The final estimation of the numbers and prevalence of pathogens in foods to be consumed is generally based on an accumulation of data throughout the food chain. In this regard, Noroviruses are detected at all production levels in all types of bivalve molluscs, but transport and storage have little influence on contamination levels [202].

In order to estimate the exposure to Norovirus through the consumption of a portion of shellfish, various factors must be known. Indeed, the overall exposure assessment relates the amount of contaminant in a designated amount of food with the amount of food typically consumed in a single serving. The EU fish market reports that 1.28 kg/per capita of mussels have been consumed in Europe in 2017 [203]. Older data are available: in Portugal 11.51 g/per capita/day in 2009 are reported, while in Spain a declining trend has been described from 2010 to 2014 when 1.21 kg of mussels pro/capita/year were consumed [204]. Similarly, in France in 2017, the average consumption was of 1.8 kg/capita/year (data comprise cephalopod). For oysters, information is more precise regarding the meal size distribution, which has been described to be 13.8 oysters per oyster meal [205], but also a half or a whole dozen [206]. While 6–12 mussels are usually set aside, no information is yet available about clams.

## 4. Hazard Characterization

Both temporal and geographical fluctuation of the concentration of the virus within molluscs have been described in the literature [207,208]. Seasonality is indeed a known characteristic of NoV illnesses because of shellfish consumption that shows a peak incidence during the wintertime. Importantly, bioaccumulation and elimination kinetics of bacteria or viruses by bivalve molluscs vary with the shellfish species, type of micro-organism and environmental conditions [6].

To date, there is no single consensus model for recommended use in risk assessment [209]. Indeed, the infective dose, or the point at which 50% of the population would become ill when exposed to the virus, is difficult to determine. Variability associated with host factors (secretor +/−) and pathogen factors (aggregated, aggregation size and non-aggregated) are important parameters.

NoV is among the most infectious agents, with low infectious odds [117,210,211,212,213], posing a substantial economic burden since it is highly transmittable and contagious [214]. However, current estimates suggest that the infective dose lies in a range between 15 and 1.300 genome copies or 1–10 virus particles [117,202]. Nevertheless, oysters with lower concentrations have been implicated in outbreaks [210]. A correlation has been found between the number of viral genome copies in bivalve shellfish and the amount of reported illness for NoV, suggesting that detection of high levels of virus RNA in shellfish is indicative of a high health risk [14,215].

## 5. Discussion

The data collected in this review allows us to identify the key data gaps to perform a full quantitative microbial risk assessment for Norovirus in bivalve molluscs. An overall summary is reported in Table 1

### 5.1. Exposure Pathways

A comprehensive discussion can be made referring to pathways # 1, #2 and #3. 

Indeed, the bivalve molluscs considered within the three pathways originate from different production chains, but in all the scenarios the product is sold with a High likelihood of the presence of the virus. This assessment should be connected with the evidence that the consumer behaviour resulted in the major risk assessment variable and one of the most important operational reference points was useful to determine appropriate risk reductions. In other words, the consumer behaviour has a greater impact than NoV prevalence, and the food safety approach depends on the variability surrounding the risk profile of the food. Indeed, the reduction of the viral load to a level corresponding to a Negligible risk is obtained only by thermal treatment, which is demanded to the consumer. Recent data obtained from experimental infection of Manila clams (*R. philippinarum*), demonstrate that when cooking by a traditional domestic method, the time needed for the majority of valves to open up can efficiently eliminate the risk [216]; this corresponds to a temperature close to 100 °C for at least 2 min. Another consideration for the consumers must be taken in relation to the storage of foods. In contrast to many microorganisms, foodborne viruses cannot grow in foods, but can survive during storage. Specifically, NoV can survive for up to 10 days in food in a refrigerator [217] and storing mussels at 4 °C revealed no reduction in NoV titers after 4 weeks [218]. Since the viral load is neither diminished nor augmented, the product after domestic storage has to be considered to present a High likelihood of the presence of the virus, and a High uncertainty.

Regarding pathway #4, we consider that the likelihood of the virus surviving to an industrial heat treatment can be considered Negligible, with a Low uncertainty.

Analysis of the fifth pathway led to the conclusion that the estimated likelihood for the presence of infectious viral particles after non-thermal treatment cannot be determined, since no data regarding outbreaks associated with non-thermal treated mussels are available to date.

### 5.2. Hazard Identification

Mollusc-mediated NoV outbreaks should be approached in terms of an important zoonotic pathology where the introduction of novel NoV strains into the human population facilitating viral recombination is possible within each outbreak. Outbreaks could be prevented by performing shellfish analysis at the times of the year at which Norovirus risk is elevated and by following microbial alert events, such as sewage overflows and heavy rainfall, as already suggested [151]. To do so, a comparison of epidemic strains and those circulating at low levels in the population, not only aiming at symptomatic cases, should be performed. In this regard, monitoring of NoV in raw sewage or treated effluents has been demonstrated to provide an early warning of an elevated risk for NoV and potentially help prevent outbreaks through environmental exposure. In case of production areas that host different shellfish types, the use of the one with the highest contamination level as an indicator for viral analysis in monitoring programs or in surveillance might be adopted [115]. Last but not least, vigilant molecular surveillance would help in developing vaccines.

To date, Norovirus detection methods have been based on a quantitative real-time PCR- ISO (15216-1:2017), which lacks standard methods for accurate quantification of infective and non-infective (damaged) NoV particles, hampering the identification of an appropriate lower NoV contamination limit for shellfish. As a consequence, proof of NoV infectivity in bivalves remains challenging, also taking into account the scarcity of data available to reliably predict illness from measured NoV levels. Optimization of diagnostic techniques should improve the understanding and the management of risk to consumers associated with the detection of NoV RNA in foodstuffs in order to better address the correlation between the presence of RNA and infectivity.

### 5.3. Exposure Assessment

Data regarding consumption of bivalve molluscs are scarce and fragmented but in general, oysters are known to be more frequently eaten raw than clams and mussels. Member states and the European Commission should perform a more detailed collection of information on annual consumption and per capita consumption, as well as on serving sizes, taking into account the different categories of consumers, especially the ones most at risk. Data should comprise information also regarding bivalve preparation in the household, particularly focusing on the preparation method in regard to cooking/non cooking methods. Indeed, scarce data are available on how consumers prepare their food, and also regarding the percentage of consumers that prefer to eat raw or undercooked bivalve molluscs. In addition, no precise information is available regarding the different species preferences between different populations. Data concerning infrequently consumed foods are more difficult to be captured with national surveys and should be obtained through targeted surveys that are thought to provide more precise information also about such characteristics as shellfish meal sizes [206].

### 5.4. Hazard Characterization

To support improved risk assessment for environmentally relevant exposures, a consensus dose-response model should be either developed or improved. Meanwhile, multiple models should be used to provide a range of predicted outcomes for the probability of infection in order to take into account the aggregated/non aggregated state of the virus, as well as its genogroup, the exposed population and the fraction of infectious to total viral particles.

## 6. Conclusions

All in all, surveillance measures should be implemented in the primary production and effort should be applied in order to identify the elements posing a major risk.

In this regard, pollution quantification, pollution prevention and/or sewage treatment processes might help reducing viral contamination in harvesting areas. Regulations regarding wastewater quality and disposal are crucial, but seem to have been insufficient thus far. Particular attention should be applied to the selection strategies of the harvesting areas, for example, with the institution of a minimum distance from fecal contamination sources, as well as the creation of buffer areas. In areas where bivalve molluscs intended for raw consumption are harvested, data collection can be mitigation-oriented, thus foreseeing the likelihood of events that can impact bivalve mollusc contamination (e.g., rainfalls, human epidemic events). Importantly, risk-based monitoring should be applied to the production of foods that constitute a major health risk, such as oysters.

Since water quality parameters are difficult to manage, post-harvest interventions are crucial in order to obtain a safe product. In this regard, more studies considering depuration and relaying parameters affecting viral elimination (time, water temperature, salinity, dissolved oxygen, and bivalve mollusc condition) should be undertaken. In addition, it should be considered that the measurement and recording of parameters in depuration plants may help to improve epidemiological studies, as well as to better understand cases of viral outbreaks associated with consumption of depurated products. Indeed, depuration is known to be insufficient for viral elimination; in addition, data on related outbreaks are missing. In general, a better understanding of the binding—or rather, of the unbinding—process of the virus to mollusc ligands represent a key point for future studies.

Following consumer demand for minimally processed food, non-thermal treatments represent an important sector where valuable alternatives for commercial development can be investigated. In particular, regarding HPP, studies should be undertaken considering not only the type of examined viral particle (HNoV or surrogates) and different strains, but also taking into account the single processing parameters (time, temperature and pressure) as well as matrix and composition (salinity and pH) and species-specific differences, always in the light of consumer acceptability.

In view of a potential NoV burden increase, as well as in order to eventually anticipate the emergence of novel variants, the implementation of epidemiological data collection (comprising standardized genotyping, collection of data concerning viral strains and comparison of sequences from outbreaks) should be performed, both at a local and on a global scale. In addition, a more detailed and systematic data collection should be performed at production areas and despatch centre levels, where no standardized reporting model is set.

Awareness and education campaigns should involve consumers, also considering the health benefits associated with bivalve consumption. Regarding bivalve consumption, the data are still poor, while detailed information is needed to perform a quantitative risk assessment.

From a laboratory perspective, studies into HNoV would benefit from the development of qPCR methods for the selective detection of infectious viruses, as well as the establishment of a threshold infectivity limit. In addition, effort for the application of NoV cell culture assays, avoiding the use of surrogates, should be put into studies on food matrices in order to further investigate infectivity, as well as inactivation kinetics. Importantly, the development of testing procedures is crucial in order to establish an acceptable limit for NoV and to develop a regulatory context. Waiting for a more comprehensive European surveillance plan, a starting point can be the implementation of national surveillance plans. In addition, awareness can be raised among veterinary competent authorities, about recording the production area in the RASFF database, in order to implement epidemiological information in the case of outbreaks.

## Figures and Tables

**Table 1:** Relationship between production area, processing, and health risk. MBM = marine bivalve molluscs.

Processing		Production Area	Health Risk	Missing/ Insufficient Data	Available Data	Instructions for Consumer	References
**No processing requested**		A	High if consumed raw Negligible if consumed cooked	Epidemiological data on the presence and quantification of NoV in MBM Epidemiological data on MBM consumption and consumers’ behaviour Consumer education on MBM consumption	Not structured data on the presence of NoV in class A MBM	To be cooked	[12,25,113,139]
**Depuration**		B	High	Guidelines on specific depuration parameters, if effective	Not structure data on the presence of NoV in depurated MBM		[11,12,13,25,97,113,115,152,153,154,155,156,157,158,159,160,161,162,163,164,165,166,167,168,169,170,171,172,173]
**Long Relaying (≥2 months)**		BC	High	Guidelines on specific relaying parameters, if effective	Not structured data on the presence of NoV in relayed MBM		[10,25,115,154,174]
**Transformation**	**Thermal treatment**	BC	Negligible	Not applicable	Data on minimum time/temperature combination for NoV inactivation	None	[175,176,177,178,179,180,181]
	**Non thermal treatment**		Not determined	Comprehensive dataset on HPP parameters, considering food characteristics Data on novel non thermal methods Data on outbreaks associated with HPP treatment	Not structured data regarding different HPP parameters, different MBM species, different starting NoV concentration	None	[182,183,184,185,186,187,188,189,190,191,192,193,194,195,196,197,198,199,200,201]

## References

[1] European Food Safety Authority, European Centre for Disease Prevention and Control (2021). The European Union One Health 2019 Zoonoses Report. EFSA J..

[2] Shah M.P., Hall A.J. (2017). Global Disease Burden of Foodborne Illnesses Associated with Norovirus. The Norovirus.

[3] Guix S., Pintó R., Bosch A. (2019). Final Consumer Options to Control and Prevent Foodborne Norovirus Infections. Viruses.

[4] Montazeri N., Goettert D., Achberger E.C., Johnson C.N., Prinyawiwatkul W., Janes M.E. (2015). Pathogenic Enteric Viruses and Microbial Indicators during Secondary Treatment of Municipal Wastewater. Appl. Environ. Microbiol..

[5] Palfrey R., Harman M., Moore R. (2011). Impact of Waste Water Treatments on Removal of Noroviruses from Sewage.

[6] Burkhardt W., Calci K.R. (2000). Selective Accumulation May Account for Shellfish-Associated Viral Illness. Appl. Environ. Microbiol..

[7] Formiga-Cruz M., Allard A.K., Conden-Hansson A.-C., Henshilwood K., Hernroth B.E., Jofre J., Lees D.N., Lucena F., Papapetropoulou M., Rangdale R.E. (2003). Evaluation of Potential Indicators of Viral Contamination in Shellfish and Their Applicability to Diverse Geographical Areas. AEM.

[8] Flannery J., Keaveney S., Doré W. (2009). Use of FRNA Bacteriophages To Indicate the Risk of Norovirus Contamination in Irish Oysters. J. Food Prot..

[9] Winterbourn J.B., Clements K., Lowther J.A., Malham S.K., McDonald J.E., Jones D.L. (2016). Use of Mytilus Edulis Biosentinels to Investigate Spatial Patterns of Norovirus and Faecal Indicator Organism Contamination around Coastal Sewage Discharges. Water Res..

[10] McLeod C., Polo D., Le Saux J.-C., Le Guyader F.S. (2017). Depuration and Relaying: A Review on Potential Removal of Norovirus from Oysters: Effectiveness of Viral Depuration. Compr. Rev. Food Sci. Food Saf..

[11] Sharp J.H., Clements K., Diggens M., McDonald J.E., Malham S.K., Jones D.L.E. (2021). Coli Is a Poor End-Product Criterion for Assessing the General Microbial Risk Posed From Consuming Norovirus Contaminated Shellfish. Front. Microbiol..

[12] Dirks R.A.M., Jansen C.C.C., Hägele G., Zwartkruis-Nahuis A.J.T., Tijsma A.S.L., Boxman I.L.A. (2021). Quantitative Levels of Norovirus and Hepatitis A Virus in Bivalve Molluscs Collected along the Food Chain in the Netherlands, 2013–2017. Int. J. Food Microbiol..

[13] EFSA Panel on Biological Hazards (BIOHAZ) (2012). Norovirus (NoV) in Oysters: Methods, Limits and Control Options. EFSA J..

[14] Lowther J.A., Gustar N.E., Powell A.L., Hartnell R.E., Lees D.N. (2012). Two-Year Systematic Study To Assess Norovirus Contamination in Oysters from Commercial Harvesting Areas in the United Kingdom. Appl. Environ. Microbiol..

[15] Gerba C.P., Goyal S.M., LaBelle R.L., Cech I., Bodgan G.F. (1979). Failure of Indicator Bacteria to Reflect the Occurrence of Enteroviruses in Marine Waters. Am. J. Public Health.

[16] Ang L.H. (1998). An Outbreak of Viral Gastroenteritis Associated with Eating Raw Oysters. Commun. Dis. Public Health.

[17] Romalde J. (2002). Prevalence of Enterovirus and Hepatitis A Virus in Bivalve Molluscs from Galicia (NW Spain): Inadequacy of the EU Standards of Microbiological Quality. Int. J. Food Microbiol..

[18] Baert L., Mattison K., Loisy-Hamon F., Harlow J., Martyres A., Lebeau B., Stals A., Van Coillie E., Herman L., Uyttendaele M. (2011). Review: Norovirus Prevalence in Belgian, Canadian and French Fresh Produce: A Threat to Human Health?. Int. J. Food Microbiol..

[19] Oh E.G., Song K.C., Kim S., Park K., Yu H. (2015). Negative Correlation between the Prevalence of Norovirus and High Bacterial Loads of Escherichia Coli in Oysters Crassostrea Gigas. Fish. Aquat. Sci..

[20] Younger A.D., Teixeira Alves M., Taylor N.G.H., Lowther J., Baker-Austin C., Campos C.J.A., Price-Hayward M., Lees D. (2018). Evaluation of the Protection against Norovirus Afforded by E. Coli Monitoring of Shellfish Production Areas under EU Regulations. Water Sci. Technol..

[21] Knight A., Li D., Uyttendaele M., Jaykus L.-A. (2013). A Critical Review of Methods for Detecting Human Noroviruses and Predicting Their Infectivity. Crit. Rev. Microbiol..

[22] Topping J.R., Schnerr H., Haines J., Scott M., Carter M.J., Willcocks M.M., Bellamy K., Brown D.W., Gray J.J., Gallimore C.I. (2009). Temperature Inactivation of Feline Calicivirus Vaccine Strain FCV F-9 in Comparison with Human Noroviruses Using an RNA Exposure Assay and Reverse Transcribed Quantitative Real-Time Polymerase Chain Reaction—A Novel Method for Predicting Virus Infectivity. J. Virol. Methods.

[23] Bosch A., Pintó R.M., Guix S. (2016). Foodborne Viruses. Curr. Opin. Food Sci..

[24] Liu L., Moore M.D. (2020). A Survey of Analytical Techniques for Noroviruses. Foods.

[25] European Food Safety Authority (EFSA) (2019). Analysis of the European Baseline Survey of Norovirus in Oysters. EFSA J..

[26] EFSA Panel on Biological Hazards (BIOHAZ) (2011). Scientific Opinion on an Update on the Present Knowledge on the Occurrence and Control of Foodborne Viruses. EFSA J..

[27] EFSA Panel on Biological Hazards (BIOHAZ) (2015). Evaluation of Heat Treatments, Different from Those Currently Established in the EU Legislation, That Could Be Applied to Live Bivalve Molluscs from B and C Production Areas, That Have Not Been Submitted to Purification or Relaying, in Order to Eliminate Pathogenic Microorganisms. EFSA J..

[28] Hassard F., Sharp J.H., Taft H., LeVay L., Harris J.P., McDonald J.E., Tuson K., Wilson J., Jones D.L., Malham S.K. (2017). Critical Review on the Public Health Impact of Norovirus Contamination in Shellfish and the Environment: A UK Perspective. Food Environ. Virol..

[29] Cann K.F., Thomas D.R., Salmon R.L., Wyn-Jones A.P., Kay D. (2013). Extreme Water-Related Weather Events and Waterborne Disease. Epidemiol. Infect..

[30] Waste Water Treatment for 21st Century Challenges. https://www.eea.europa.eu/publications/urban-waste-water-treatment-for.

[31] Lipp E.K., Kurz R., Vincent R., Rodriguez-Palacios C., Farrah S.R., Rose J.B. (2001). The Effects of Seasonal Variability and Weather on Microbial Fecal Pollution and Enteric Pathogens in a Subtropical Estuary. Estuaries.

[32] Griffin D.W., Donaldson K.A., Paul J.H., Rose J.B. (2003). Pathogenic Human Viruses in Coastal Waters. CMR.

[33] Le Guyader F., Loisy F., Atmar R.L., Hutson A.M., Estes M.K., Ruvoën-Clouet N., Pommepuy M., Le Pendu J. (2006). Norwalk Virus-Specific Binding to Oyster Digestive Tissues. Emerg. Infect. Dis..

[34] Riou P., Le Saux J.C., Dumas F., Caprais M.P., Le Guyader S.F., Pommepuy M. (2007). Microbial Impact of Small Tributaries on Water and Shellfish Quality in Shallow Coastal Areas. Water Res..

[35] Coulliette A.D., Money E.S., Serre M.L., Noble R.T. (2009). Space/Time Analysis of Fecal Pollution and Rainfall in an Eastern North Carolina Estuary. Environ. Sci. Technol..

[36] Campos C.J.A., Kershaw S.R., Lee R.J. (2013). Environmental Influences on Faecal Indicator Organisms in Coastal Waters and Their Accumulation in Bivalve Shellfish. Estuaries Coasts.

[37] Campos C.J.A., Lees D.N. (2014). Environmental Transmission of Human Noroviruses in Shellfish Waters. Appl. Environ. Microbiol..

[38] Miossec L., Le Guyader F., Haugarreau L., Comps M.A., Pommepuy M. (1998). Possible Relation between a Winter Epidemic of Acute Gastroenteritis in France and Viral Contamination of Shellfish. J. Shellfish Res..

[39] Yates M.V. (1985). Septic Tank Density and Ground-Water Contamination. Ground Water.

[40] Madan N.J., Marshall W.A., Laybourn-Parry J. (2005). Virus and Microbial Loop Dynamics over an Annual Cycle in Three Contrasting Antarctic Lakes. Freshw. Biol..

[41] Lymer D., Vrede K. (2006). Nutrient Additions Resulting in Phage Release and Formation of Non-Nucleoid-Containing Bacteria. Aquat. Microb. Ecol..

[42] Fallahi S., Mattison K. (2011). Evaluation of Murine Norovirus Persistence in Environments Relevant to Food Production and Processing. J. Food Prot..

[43] Gantzer C., Dubois É., Crance J.-M., Billaudel S., Kopecka H., Schwartzbrod L., Pommepuy M., Guyader F.L. (1998). Devenir des virus entériques en mer et influence des facteurs environnementaux. Oceanol. Acta.

[44] Bitton G. (1999). Wastewater Microbiology.

[45] Bae J., Schwab K.J. (2008). Evaluation of Murine Norovirus, Feline Calicivirus, Poliovirus, and MS2 as Surrogates for Human Norovirus in a Model of Viral Persistence in Surface Water and Groundwater. AEM.

[46] Ngazoa E.S., Fliss I., Jean J. (2008). Quantitative Study of Persistence of Human Norovirus Genome in Water Using TaqMan Real-Time RT-PCR. J. Appl. Microbiol..

[47] Skraber S., Ogorzaly L., Helmi K., Maul A., Hoffmann L., Cauchie H.-M., Gantzer C. (2009). Occurrence and Persistence of Enteroviruses, Noroviruses and F-Specific RNA Phages in Natural Wastewater Biofilms. Water Res..

[48] Liu P., Jaykus L.-A., Wong E., Moe C. (2012). Persistence of Norwalk Virus, Male-Specific Coliphage, and Escherichia Coli on Stainless Steel Coupons and in Phosphate-Buffered Saline. J. Food Prot..

[49] Kauppinen A., Miettinen I. (2017). Persistence of Norovirus GII Genome in Drinking Water and Wastewater at Different Temperatures. Pathogens.

[50] John D.E., Rose J.B. (2005). Review of Factors Affecting Microbial Survival in Groundwater. Environ. Sci. Technol..

[51] Gerba C.P. (2007). Virus Occurrence and Survival in the Environmental Waters. Perspectives in Medical Virology.

[52] Lopman B., Armstrong B., Atchison C., Gray J.J. (2009). Host, Weather and Virological Factors Drive Norovirus Epidemiology: Time-Series Analysis of Laboratory Surveillance Data in England and Wales. PLoS ONE.

[53] Seitz S.R., Leon J.S., Schwab K.J., Lyon G.M., Dowd M., McDaniels M., Abdulhafid G., Fernandez M.L., Lindesmith L.C., Baric R.S. (2011). Norovirus Infectivity in Humans and Persistence in Water. Appl. Environ. Microbiol..

[54] Nordgren J., Matussek A., Mattsson A., Svensson L., Lindgren P.-E. (2009). Prevalence of Norovirus and Factors Influencing Virus Concentrations during One Year in a Full-Scale Wastewater Treatment Plant. Water Res..

[55] Summa M., von Bonsdorff C.-H., Maunula L. (2012). Pet Dogs—A Transmission Route for Human Noroviruses?. J. Clin. Virol..

[56] Charoenkul K., Nasamran C., Janetanakit T., Tangwangvivat R., Bunpapong N., Boonyapisitsopa S., Suwannakarn K., Theamboonler A., Chuchaona W., Poovorawan Y. (2020). Human Norovirus Infection in Dogs, Thailand. Emerg. Infect. Dis..

[57] Villabruna N., Schapendonk C.M.E., Aron G.I., Koopmans M.P.G., de Graaf M. (2020). Human Noroviruses Attach to Intestinal Tissues of a Broad Range of Animal Species. J. Virol..

[58] Widdowson M.-A., Rockx B., Schepp R., van der Poel W.H.M., Vinje J., van Duynhoven Y.T., Koopmans M.P. (2005). Detection of Serum Antibodies to Bovine Norovirus in Veterinarians and the General Population in the Netherlands. J. Med. Virol..

[59] Vildevall M., Grahn A., Oliver S.L., Bridger J.C., Charpilienne A., Poncet D., Larson G., Svensson L. (2010). Human Antibody Responses to Bovine (Newbury-2) Norovirus (GIII.2) and Association to Histo-Blood Group Antigens. J. Med. Virol..

[60] Menon V.K., George S., Shanti A.A., Saravanabavan A., Samuel P., Ramani S., Estes M.K., Kang G. (2013). Exposure to Human and Bovine Noroviruses in a Birth Cohort in Southern India from 2002 to 2006. J. Clin. Microbiol..

[61] Mesquita J., Costantini V.P., Cannon J.L., Lin S., Nascimento M.S., Vinjé J. (2013). Presence of Antibodies against Genogroup VI Norovirus in Humans. Virol. J..

[62] Di Martino B., Di Profio F., Ceci C., Di Felice E., Green K.Y., Bok K., De Grazia S., Giammanco G.M., Massirio I., Lorusso E. (2014). Seroprevalence of Norovirus Genogroup IV Antibodies among Humans, Italy, 2010–2011. Emerg. Infect. Dis..

[63] Chan M.C.W., Shan Kwan H., Chan P.K.S. (2017). Structure and Genotypes of Noroviruses. The Norovirus.

[64] Lysen M., Thorhagen M., Brytting M., Hjertqvist M., Andersson Y., Hedlund K.-O. (2009). Genetic Diversity among Food-Borne and Waterborne Norovirus Strains Causing Outbreaks in Sweden. J. Clin. Microbiol..

[65] De Graaf M., van Beek J., Koopmans M.P.G. (2016). Human Norovirus Transmission and Evolution in a Changing World. Nat. Rev. Microbiol..

[66] Rajko-Nenow P., Waters A., Keaveney S., Flannery J., Tuite G., Coughlan S., O’Flaherty V., Doré W. (2013). Norovirus Genotypes Present in Oysters and in Effluent from a Wastewater Treatment Plant during the Seasonal Peak of Infections in Ireland in 2010. Appl. Environ. Microbiol..

[67] Ludwig-Begall L.F., Mauroy A., Thiry E. (2018). Norovirus Recombinants: Recurrent in the Field, Recalcitrant in the Lab—A Scoping Review of Recombination and Recombinant Types of Noroviruses. J. Gen. Virol..

[68] Bull R.A., Eden J.-S., Rawlinson W.D., White P.A. (2010). Rapid Evolution of Pandemic Noroviruses of the GII.4 Lineage. PLoS Pathog..

[69] Liu B.L., Lambden P.R., Günther H., Otto P., Elschner M., Clarke I.N. (1999). Molecular Characterization of a Bovine Enteric Calicivirus: Relationship to the Norwalk-Like Viruses. J. Virol..

[70] Karst S.M. (2003). STAT1-Dependent Innate Immunity to a Norwalk-Like Virus. Science.

[71] Wang Q.-H., Han M.G., Cheetham S., Souza M., Funk J.A., Saif L.J. (2005). Porcine Noroviruses Related to Human Noroviruses. Emerg. Infect. Dis..

[72] Wolf S., Williamson W., Hewitt J., Lin S., Rivera-Aban M., Ball A., Scholes P., Savill M., Greening G.E. (2009). Molecular Detection of Norovirus in Sheep and Pigs in New Zealand Farms. Vet. Microbiol..

[73] Mesquita J.R., Barclay L., Nascimento M.S.J., Vinjé J. (2010). Novel Norovirus in Dogs with Diarrhea. Emerg. Infect. Dis..

[74] Shen Q., Zhang W., Yang S., Cui L., Hua X. (2012). Complete Genome Sequence of a New-Genotype Porcine Norovirus Isolated from Piglets with Diarrhea. J. Virol..

[75] Knipe D.M., Howley P.M. (2013). Fields Virology.

[76] Di Martino B., Di Profio F., Melegari I., Sarchese V., Cafiero M.A., Robetto S., Aste G., Lanave G., Marsilio F., Martella V. (2016). A Novel Feline Norovirus in Diarrheic Cats. Infect. Genet. Evol..

[77] Chhabra P., de Graaf M., Parra G.I., Chan M.C.-W., Green K., Martella V., Wang Q., White P.A., Katayama K., Vennema H. (2019). Updated Classification of Norovirus Genogroups and Genotypes. J. Gen. Virol..

[78] Hall A.J. (2012). Noroviruses: The Perfect Human Pathogens?. J. Infect. Dis..

[79] European Food Safety Authority, European Centre for Disease Prevention and Control (2013). The European Union Summary Report on Trends and Sources of Zoonoses, Zoonotic Agents and Food-borne Outbreaks in 2011. EFSA J..

[80] Gould L.H., Walsh K.A., Vieira A.R., Herman K., Williams I.T., Hall A.J., Cole D. (2013). Centers for Disease Control and Prevention Surveillance for Foodborne Disease Outbreaks—United States, 1998–2008. MMWR Surveill. Summ..

[81] Venugopal V., Gopakumar K. (2017). Shellfish: Nutritive Value, Health Benefits, and Consumer Safety: Shellfish Nutritive Value and Safety. Compr. Rev. Food Sci. Food Saf..

[82] Ozawa H., Kumazaki M., Ueki S., Morita M., Usuku S. (2015). Detection and Genetic Analysis of Noroviruses and Sapoviruses in Sea Snail. Food Environ. Virol..

[83] Technical Report: Review of Quantitative Risk Assessment of Foodborne Norovirus Transmission. https://www.food.gov.uk/sites/default/files/media/document/technical-report-review-of-quantitative-risk-assessment-of-norovirus-transmission_0.pdf.

[84] Richards G.P. (1988). Microbial Purification of Shellfish: A Review of Depuration and Relaying. J. Food Prot..

[85] Atmar R.L., Neill F.H., Romalde J.L., Le Guyader F., Woodley C.M., Metcalf T.G., Estes M.K. (1995). Detection of Norwalk Virus and Hepatitis A Virus in Shellfish Tissues with the PCR. Appl. Environ. Microbiol..

[86] Le Guyader F.S., Atmar R.L., Le Pendu J. (2012). Transmission of Viruses through Shellfish: When Specific Ligands Come into Play. Curr. Opin. Virol..

[87] Yu Y., Cai H., Hu L., Lei R., Pan Y., Yan S., Wang Y. (2015). Molecular Epidemiology of Oyster-Related Human Noroviruses and Their Global Genetic Diversity and Temporal-Geographical Distribution from 1983 to 2014. Appl. Environ. Microbiol..

[88] Mcleod C., Hay B., Grant C., Greening G., Day D. (2009). Localization of Norovirus and Poliovirus in Pacific Oysters. J. Appl. Microbiol..

[89] Comelli H., Rimstad E., Larsen S., Myrmel M. (2008). Detection of Norovirus Genotype I.3b and II.4 in Bioaccumulated Blue Mussels Using Different Virus Recovery Methods. Int. J. Food Microbiol..

[90] Suffredini E., Pepe T., Ventrone I., Croci L. (2011). Norovirus Detection in Shellfish Using Two Real-Time RT-PCR Methods. New Microbiol..

[91] Siebenga J.J., Vennema H., Zheng D., Vinjé J., Lee B.E., Pang X., Ho E.C.M., Lim W., Choudekar A., Broor S. (2009). Norovirus Illness Is a Global Problem: Emergence and Spread of Norovirus GII.4 Variants, 2001–2007. J. Infect. Dis..

[92] Maalouf H., Zakhour M., Le Pendu J., Le Saux J.-C., Atmar R.L., Le Guyader F.S. (2010). Distribution in Tissue and Seasonal Variation of Norovirus Genogroup I and II Ligands in Oysters. AEM.

[93] Diez-Valcarce M., Kokkinos P., Söderberg K., Bouwknegt M., Willems K., de Roda-Husman A.M., von Bonsdorff C.-H., Bellou M., Hernández M., Maunula L. (2012). Occurrence of Human Enteric Viruses in Commercial Mussels at Retail Level in Three European Countries. Food Environ. Virol..

[94] Rajko-Nenow P., Keaveney S., Flannery J., O’Flaherty V., Doré W. (2012). Characterisation of Norovirus Contamination in an Irish Shellfishery Using Real-Time RT-QPCR and Sequencing Analysis. Int. J. Food Microbiol..

[95] Razafimahefa R.M., Ludwig-Begall L.F., Thiry E. (2020). *Cockles and Mussels, Alive, Alive, Oh*—The Role of Bivalve Molluscs as Transmission Vehicles for Human Norovirus Infections. Transbound. Emerg. Dis..

[96] Gabrieli R., Macaluso A., Lanni L., Saccares S., Di Giamberardino F., Cencioni B., Petrinca A.R., Divizia M. (2007). Enteric Viruses in Molluscan Shellfish. New Microbiol..

[97] Savini G., Casaccia C., Barile N.B., Paoletti M., Pinoni C. (2009). Norovirus in Bivalve Molluscs: A Study of the Efficacy of the Depuration System. Vet. Ital..

[98] Moreno E., Espigares E., Marañón M., Ochoa L.M., Espigares M., Fernández-Crehuet M. (2014). The Prevalence of Noroviruses in Bivalve Molluscs Sold in Granada (Spain) Fish Markets. Molluscan Res..

[99] Hansman G.S., Oka T., Li T.-C., Nishio O., Noda M., Takeda N. (2008). Detection of Human Enteric Viruses in Japanese Clams. J. Food Prot..

[100] Parra G.I. (2019). Emergence of Norovirus Strains: A Tale of Two Genes. Virus Evol..

[101] Noel J.S., Fankhauser R.L., Ando T., Monroe S.S., Glass R.I. (1999). Identification of a Distinct Common Strain of “Norwalk-like Viruses” Having a Global Distribution. J. Infect. Dis..

[102] Van Beek J., Ambert-Balay K., Botteldoorn N., Eden J.S., Fonager J., Hewitt J., Iritani N., Kroneman A., Vennema H., Vinjé J. (2013). Indications for Worldwide Increased Norovirus Activity Associated with Emergence of a New Variant of Genotype II.4, Late 2012. Eurosurveillance.

[103] Eden J.-S., Hewitt J., Lim K.L., Boni M.F., Merif J., Greening G., Ratcliff R.M., Holmes E.C., Tanaka M.M., Rawlinson W.D. (2014). The Emergence and Evolution of the Novel Epidemic Norovirus GII.4 Variant Sydney 2012. Virology.

[104] De Graaf M., van Beek J., Vennema H., Podkolzin A.T., Hewitt J., Bucardo F., Templeton K., Mans J., Nordgren J., Reuter G. (2015). Emergence of a Novel GII.17 Norovirus—End of the GII.4 Era?. Eurosurveillance.

[105] Iritani N., Yamamoto S.P., Abe N., Kanbayashi D., Kubo H., Uema M., Noda M., Kaida A. (2019). GII.17 Norovirus Infections in Outbreaks of Acute Nonbacterial Gastroenteritis in Osaka City, Japan during Two Decades. J. Med. Virol..

[106] Lu J., Sun L., Fang L., Yang F., Mo Y., Lao J., Zheng H., Tan X., Lin H., Rutherford S. (2015). Gastroenteritis Outbreaks Caused by Norovirus GII.17, Guangdong Province, China, 2014–2015. Emerg. Infect. Dis..

[107] Parra G.I., Green K.Y. (2015). Genome of Emerging Norovirus GII.17, United States, 2014. Emerg. Infect. Dis..

[108] Lee C.-C., Feng Y., Chen S.-Y., Tsai C.-N., Lai M.-W., Chiu C.-H. (2015). Emerging Norovirus GII.17 in Taiwan. Clin. Infect. Dis..

[109] Medici M.C., Tummolo F., Calderaro A., Chironna M., Giammanco G.M., De Grazia S., Arcangeletti M.C., De Conto F., Chezzi C., Martella V. (2015). Identification of the Novel Kawasaki 2014 GII.17 Human Norovirus Strain in Italy, 2015. Eurosurveillance.

[110] Chan M.C.W., Lee N., Hung T.-N., Kwok K., Cheung K., Tin E.K.Y., Lai R.W.M., Nelson E.A.S., Leung T.F., Chan P.K.S. (2015). Rapid Emergence and Predominance of a Broadly Recognizing and Fast-Evolving Norovirus GII.17 Variant in Late 2014. Nat. Commun..

[111] Andrade J.S.R., Fumian T.M., Leite J.P.G., Assis M.R.d., Bello G., Mir D., Miagostovich M.P. (2017). Detection and Molecular Characterization of Emergent GII.P17/GII.17 Norovirus in Brazil, 2015. Infect. Genet. Evol..

[112] La Rosa G., Della Libera S., Iaconelli M., Proroga Y.T.R., De Medici D., Martella V., Suffredini E. (2017). Detection of Norovirus GII.17 Kawasaki 2014 in Shellfish, Marine Water and Underwater Sewage Discharges in Italy. Food Environ. Virol..

[113] Suffredini E., Lanni L., Arcangeli G., Pepe T., Mazzette R., Ciccaglioni G., Croci L. (2014). Qualitative and Quantitative Assessment of Viral Contamination in Bivalve Molluscs Harvested in Italy. Int. J. Food Microbiol..

[114] Polo D., Varela M.F., Romalde J.L. (2015). Detection and Quantification of Hepatitis A Virus and Norovirus in Spanish Authorized Shellfish Harvesting Areas. Int. J. Food Microbiol..

[115] Suffredini E., Magnabosco C., Civettini M., Rossetti E., Arcangeli G., Croci L. (2012). Norovirus Contamination in Different Shellfish Species Harvested in the Same Production Areas. J. Appl. Microbiol..

[116] Zhao F., Ding G., Wang S., Cai Y., Xu J., Cheng J., Zhou D. (2021). Preliminary Quantitative Risk Assessment of Norovirus in Shellfish in the Yellow Sea and Bohai Sea of China. Foodborne Pathog. Dis..

[117] Teunis P.F.M., Moe C.L., Liu P., Miller S.E., Lindesmith L., Baric R.S., Le Pendu J., Calderon R.L. (2008). Norwalk Virus: How Infectious Is It?. J. Med. Virol..

[118] Le Pendu J., Nyström K., Ruvoën-Clouet N. (2014). Host–Pathogen Co-Evolution and Glycan Interactions. Curr. Opin. Virol..

[119] Koopmans M. (2008). Progress in Understanding Norovirus Epidemiology. Curr. Opin. Infect. Dis..

[120] Wyatt R.G., Dolin R., Blacklow N.R., DuPont H.L., Buscho R.F., Thornhill T.S., Kapikian A.Z., Chanock R.M. (1974). Comparison of Three Agents of Acute Infectious Nonbacterial Gastroenteritis by Cross-Challenge in Volunteers. J. Infect. Dis..

[121] Kaplan J.E., Feldman R., Campbell D.S., Lookabaugh C., Gary G.W. (1982). The Frequency of a Norwalk-like Pattern of Illness in Outbreaks of Acute Gastroenteritis. Am. J. Public Health.

[122] Teunis P.F.M., Sukhrie F.H.A., Vennema H., Bogerman J., Beersma M.F.C., Koopmans M.P.G. (2015). Shedding of Norovirus in Symptomatic and Asymptomatic Infections. Epidemiol. Infect..

[123] Höhne M., Schreier E. (2004). Detection and Characterization of Norovirus Outbreaks in Germany: Application of a One-Tube RT-PCR Using a Fluorogenic Real-Time Detection System: One-Tube Real-Time PCR for Norovirus Detection. J. Med. Virol..

[124] Chan M.C.W., Sung J.J.Y., Lam R.K.Y., Chan P.K.S., Lee N.L.S., Lai R.W.M., Leung W.K. (2006). Fecal Viral Load and Norovirus-Associated Gastroenteritis. Emerg. Infect. Dis..

[125] Ozawa K., Oka T., Takeda N., Hansman G.S. (2007). Norovirus Infections in Symptomatic and Asymptomatic Food Handlers in Japan. J. Clin. Microbiol..

[126] Atmar R.L., Opekun A.R., Gilger M.A., Estes M.K., Crawford S.E., Neill F.H., Graham D.Y. (2008). Norwalk Virus Shedding after Experimental Human Infection. Emerg. Infect. Dis..

[127] Siebenga J.J., Beersma M.F.C., Vennema H., van Biezen P., Hartwig N.J., Koopmans M. (2008). High Prevalence of Prolonged Norovirus Shedding and Illness among Hospitalized Patients: A Model for In Vivo Molecular Evolution. J. Infect. Dis..

[128] Campos C.J.A., Avant J., Lowther J., Till D., Lees D.N. (2016). Human Norovirus in Untreated Sewage and Effluents from Primary, Secondary and Tertiary Treatment Processes. Water Res..

[129] Campos C.J.A., Avant J., Lowther J., Till D., Lees D. (2013). Levels of Norovirus and E. coli in Untreated, Biologically Treated and UV-Disinfected Sewage Effluent Discharged to a Shellfish Water. J. Water Resour. Prot..

[130] Pouillot R., Van Doren J.M., Woods J., Plante D., Smith M., Goblick G., Roberts C., Locas A., Hajen W., Stobo J. (2015). Meta-Analysis of the Reduction of Norovirus and Male-Specific Coliphage Concentrations in Wastewater Treatment Plants. Appl. Environ. Microbiol..

[131] Cook N., Knight A., Richards G.P. (2016). Persistence and Elimination of Human Norovirus in Food and on Food Contact Surfaces: A Critical Review. J. Food Prot..

[132] Haas C.N., Joffe J., Anmangandla U., Jacangelo J.G., Heath M. (1996). Water Quality and Disinfection Kinetics. J.-Am. Water Work. Assoc..

[133] Thurston-Enriquez J.A., Haas C.N., Jacangelo J., Gerba C.P. (2003). Chlorine Inactivation of Adenovirus Type 40 and Feline Calicivirus. Appl. Environ. Microbiol..

[134] Kahler A.M., Cromeans T.L., Roberts J.M., Hill V.R. (2011). Source Water Quality Effects on Monochloramine Inactivation of Adenovirus, Coxsackievirus, Echovirus, and Murine Norovirus. Water Res..

[135] Shin G.-A., Sobsey M.D. (2008). Inactivation of Norovirus by Chlorine Disinfection of Water. Water Res..

[136] Kroneman A., Verhoef L., Harris J., Vennema H., Duizer E., van Duynhoven Y., Gray J., Iturriza M., Bottiger B., Falkenhorst G. (2008). Analysis of Integrated Virological and Epidemiological Reports of Norovirus Outbreaks Collected within the Foodborne Viruses in Europe Network from 1 July 2001 to 30 June 2006. J. Clin. Microbiol..

[137] Pavoni E., Consoli M., Suffredini E., Arcangeli G., Serracca L., Battistini R., Rossini I., Croci L., Losio M.N. (2013). Noroviruses in Seafood: A 9-Year Monitoring in Italy. Foodborne Pathog. Dis..

[138] La Bella G., Martella V., Basanisi M.G., Nobili G., Terio V., La Salandra G. (2017). Food-Borne Viruses in Shellfish: Investigation on Norovirus and HAV Presence in Apulia (SE Italy). Food Environ. Virol..

[139] Hernroth B.E., Conden-Hansson A.-C., Rehnstam-Holm A.-S., Girones R., Allard A.K. (2002). Environmental Factors Influencing Human Viral Pathogens and Their Potential Indicator Organisms in the Blue Mussel, Mytilus Edulis: The First Scandinavian Report. AEM.

[140] Rippey S.R. (1994). Infectious Diseases Associated with Molluscan Shellfish Consumption. Clin. Microbiol. Rev..

[141] Rohayem J. (2009). Norovirus Seasonality and the Potential Impact of Climate Change. Clin. Microbiol. Infect..

[142] Myrmel M., Berg E.M.M., Grinde B., Rimstad E. (2006). Enteric Viruses in Inlet and Outlet Samples from Sewage Treatment Plants. J. Water Health.

[143] Katayama H., Haramoto E., Oguma K., Yamashita H., Tajima A., Nakajima H., Ohgaki S. (2008). One-Year Monthly Quantitative Survey of Noroviruses, Enteroviruses, and Adenoviruses in Wastewater Collected from Six Plants in Japan. Water Res..

[144] Sima L.C., Schaeffer J., Le Saux J.-C., Parnaudeau S., Elimelech M., Le Guyader F.S. (2011). Calicivirus Removal in a Membrane Bioreactor Wastewater Treatment Plant. Appl. Environ. Microbiol..

[145] Victoria M., Guimarães F.R., Fumian T.M., Ferreira F.F.M., Vieira C.B., Shubo T., Leite J.P.G., Miagostovich M.P. (2010). One Year Monitoring of Norovirus in a Sewage Treatment Plant in Rio de Janeiro, Brazil. J. Water Health.

[146] Westrell T., Teunis P., van den Berg H., Lodder W., Ketelaars H., Stenström T.A., de Roda Husman A.M. (2006). Short- and Long-Term Variations of Norovirus Concentrations in the Meuse River during a 2-Year Study Period. Water Res..

[147] Pérez-Sautu U., Sano D., Guix S., Kasimir G., Pintó R.M., Bosch A. (2012). Human Norovirus Occurrence and Diversity in the Llobregat River Catchment, Spain: Norovirus Occurrence and Diversity in Water. Environ. Microbiol..

[148] Katayama H., Tanaka A., Otaki M., Ohgaki S. (2004). Determination of Naturally Occurring Noroviruses in Coastal Seawater by Alkaline Elution after Acid Rinse Using Negatively Charged Membrane. Water Supply.

[149] Bazzardi R. (2013). Indagine Sulla Presenza di Norovirus in Molluschi Bivalvi Vivi Allevati e Commercializzati Nella Regione Sardegna. Ph.D. Thesis.

[150] Anacleto P., Barrento S., Nunes M.L., Rosa R., Marques A. (2014). Portuguese Consumers’ Attitudes and Perceptions of Bivalve Molluscs. Food Control.

[151] Polo D., Schaeffer J., Fournet N., Le Saux J.-C., Parnaudeau S., McLeod C., Le Guyader F.S. (2016). Digital PCR for Quantifying Norovirus in Oysters Implicated in Outbreaks, France. Emerg. Infect. Dis..

[152] Schwab K.J., Neill F.H., Estes M.K., Metcalf T.G., Atmar R.L. (1998). Distribution of Norwalk Virus within Shellfish Following Bioaccumulation and Subsequent Depuration by Detection Using RT-PCR. J. Food Prot..

[153] Lees D. (2000). Viruses and Bivalve Shellfish. Int. J. Food Microbiol..

[154] Richards G.P., McLeod C., Le Guyader F.S. (2010). Processing Strategies to Inactivate Enteric Viruses in Shellfish. Food Environ. Virol..

[155] Rupnik A., Doré W., Devilly L., Fahy J., Fitzpatrick A., Schmidt W., Hunt K., Butler F., Keaveney S. (2021). Evaluation of Norovirus Reduction in Environmentally Contaminated Pacific Oysters During Laboratory Controlled and Commercial Depuration. Food Environ. Virol..

[156] McLeod C., Polo D., Le Saux J.-C., Le Guyader F.S. Final Report: Evaluating the Effectiveness of Depuration in Removing Norovirus from Oysters 2017. Seafood Safety Assessment Ltd. and the 2 French Research Institute for Exploitation of the Sea. https://www.food.gov.uk/sites/default/files/media/document/fs101068finrep_0.pdf.

[157] Love D.C., Lovelace G.L., Sobsey M.D. (2010). Removal of Escherichia Coli, Enterococcus Fecalis, Coliphage MS2, Poliovirus, and Hepatitis A Virus from Oysters (Crassostrea Virginica) and Hard Shell Clams (Mercinaria Mercinaria) by Depuration. Int. J. Food Microbiol..

[158] Provost K., Dancho B.A., Ozbay G., Anderson R.S., Richards G.P., Kingsley D.H. (2011). Hemocytes Are Sites of Enteric Virus Persistence within Oysters. Appl. Environ. Microbiol..

[159] Polo D., Álvarez C., Vilariño M.L., Longa Á., Romalde J.L. (2014). Depuration Kinetics of Hepatitis A Virus in Clams. Food Microbiol..

[160] Polo D., Feal X., Romalde J.L. (2015). Mathematical Model for Viral Depuration Kinetics in Shellfish: An Useful Tool to Estimate the Risk for the Consumers. Food Microbiol..

[161] Schneider K.R., Cevallos J., Rodrick G.E. (2009). Molluscan shellfish depuration. Shellfish Safety and Quality.

[162] Neilson B. (1978). Bacterial Depuration by the American Oyster (*Crassostrea virginica*) under Controlled Conditions. Vol. 2. Practical Considerations and Plant Design.

[163] Riisgård H.U., Lüskow F., Pleissner D., Lundgreen K., López M.Á.P. (2013). Effect of Salinity on Filtration Rates of Mussels Mytilus Edulis with Special Emphasis on Dwarfed Mussels from the Low-Saline Central Baltic Sea. Helgol. Mar. Res..

[164] Borsuk M.E., Powers S.P., Peterson C.H. (2002). A Survival Model of the Effects of Bottom-Water Hypoxia on the Population Density of an Estuarine Clam (*Macoma Balthica*). Can. J. Fish. Aquat. Sci..

[165] Li Q., Sun S., Zhang F., Wang M., Li M. (2019). Effects of Hypoxia on Survival, Behavior, Metabolism and Cellular Damage of Manila Clam (Ruditapes Philippinarum). PLoS ONE.

[166] Chen J., Mai K., Ma H., Wang X., Deng D., Liu X., Xu W., Liufu Z., Zhang W., Tan B. (2007). Effects of Dissolved Oxygen on Survival and Immune Responses of Scallop (Chlamys Farreri Jones et Preston). Fish Shellfish Immunol..

[167] Polo D., Feal X., Varela M.F., Monteagudo A., Romalde J.L. (2014). Depuration Kinetics of Murine Norovirus in Shellfish. Food Res. Int..

[168] Berti M., Teodori L., Portanti O., Leone A., CARMINE I., Ferri N., Visciano P., Schirone M., Savini G. (2020). Experimental Contaminatio of Chamelea Gallina with Murine Norovirus and Effectiveness of Depuration. Ital. J. Food Sci..

[169] Pepe T., Ventrone I., Suffredini E., Ceruso M., Croci L., Anastasio A., Cortesi M.L. (2012). Norovirus Monitoring in Bivalve Molluscs Harvested and Commercialized in Southern Italy. J. Food Prot..

[170] Battistini R., Listorti V., Squadrone S., Pederiva S., Abete M.C., Mua R., Ciccotelli V., Suffredini E., Maurella C., Baioni E. (2021). Occurrence and Persistence of Enteric Viruses, Arsenic and Biotoxins in Pacific Oysters Farmed in an Italian Production Site. Mar. Pollut. Bull..

[171] Ueki Y., Shoji M., Suto A., Tanabe T., Okimura Y., Kikuchi Y., Saito N., Sano D., Omura T. (2007). Persistence of Caliciviruses in Artificially Contaminated Oysters during Depuration. Appl. Environ. Microbiol..

[172] Nappier S.P., Graczyk T.K., Schwab K.J. (2008). Bioaccumulation, Retention, and Depuration of Enteric Viruses by Crassostrea Virginica and Crassostrea Ariakensis Oysters. AEM.

[173] Younger A.D., Neish A., Walker D.I., Jenkins K.L., Lowther J.A., Stapleton T.A., Alves M.T. (2020). Strategies to Reduce Norovirus (NoV) Contamination from Oysters under Depuration Conditions. Food Chem. Toxicol..

[174] Rees G., World Health Organization (2010). Safe Management of Shellfish and Harvest Waters.

[175] Bozkurt H., D’Souza D.H., Davidson P.M. (2015). Thermal Inactivation of Foodborne Enteric Viruses and Their Viral Surrogates in Foods. J. Food Prot..

[176] Hirneisen K.A., Kniel K.E. (2013). Comparing Human Norovirus Surrogates: Murine Norovirus and Tulane Virus. J. Food Prot..

[177] Shao L., Chen H., Hicks D., Wu C. (2018). Thermal Inactivation of Human Norovirus Surrogates in Oyster Homogenate. Int. J. Food Microbiol..

[178] Ailavadi S., Davidson P.M., Morgan M.T., D’Souza D.H. (2019). Thermal Inactivation Kinetics of Tulane Virus in Cell-Culture Medium and Spinach: Heat Inactivation Kinetics of Tulane Virus. J. Food Sci..

[179] Bozkurt H., D’Souza D.H., Davidson P.M. (2013). Determination of the Thermal Inactivation Kinetics of the Human Norovirus Surrogates, Murine Norovirus and Feline Calicivirus. J. Food Prot..

[180] Bozkurt H., D’Souza D.H., Davidson P.M. (2014). A Comparison of the Thermal Inactivation Kinetics of Human Norovirus Surrogates and Hepatitis A Virus in Buffered Cell Culture Medium. Food Microbiol..

[181] Messens W., Fernandez-Escamez P.S., Lees D., Lindqvist R., O’Mahony M., Suffredini E., Cortiñas Abrahantes J., Chantzis E., Koutsoumanis K. (2018). Thermal Processing of Live Bivalve Molluscs for Controlling Viruses: On the Need for a Risk-Based Design. Crit. Rev. Food Sci. Nutr..

[182] López-Caballero M.E., Pérez-Mateos M., Montero P., Borderías A.J. (2000). Oyster Preservation by High-Pressure Treatment. J. Food Prot..

[183] Cruz-Romero M., Smiddy M., Hill C., Kerry J.P., Kelly A.L. (2004). Effects of High Pressure Treatment on Physicochemical Characteristics of Fresh Oysters (Crassostrea Gigas). Innov. Food Sci. Emerg. Technol..

[184] Kingsley D. (2014). High Pressure Processing of Bivalve Shellfish and HPP’s Use as a Virus Intervention. Foods.

[185] Lou F., Neetoo H., Chen H., Li J. (2011). Inactivation of a Human Norovirus Surrogate by High-Pressure Processing: Effectiveness, Mechanism, and Potential Application in the Fresh Produce Industry. Appl. Environ. Microbiol..

[186] Kingsley D.H., Holliman D.R., Calci K.R., Chen H., Flick G.J. (2007). Inactivation of a Norovirus by High-Pressure Processing. Appl. Environ. Microbiol..

[187] Li X., Ye M., Neetoo H., Golovan S., Chen H. (2013). Pressure Inactivation of Tulane Virus, a Candidate Surrogate for Human Norovirus and Its Potential Application in Food Industry. Int. J. Food Microbiol..

[188] Arcangeli G., Terregino C., De Benedictis P., Zecchin B., Manfrin A., Rossetti E., Magnabosco C., Mancin M., Brutti A. (2012). Effect of High Hydrostatic Pressure on Murine Norovirus in Manila Clams: HHP Effect on MNV in Manila Clams. Lett. Appl. Microbiol..

[189] Leon J.S., Kingsley D.H., Montes J.S., Richards G.P., Lyon G.M., Abdulhafid G.M., Seitz S.R., Fernandez M.L., Teunis P.F., Flick G.J. (2011). Randomized, Double-Blinded Clinical Trial for Human Norovirus Inactivation in Oysters by High Hydrostatic Pressure Processing. Appl. Environ. Microbiol..

[190] Lou F., Huang P., Neetoo H., Gurtler J.B., Niemira B.A., Chen H., Jiang X., Li J. (2012). High-Pressure Inactivation of Human Norovirus Virus-Like Particles Provides Evidence That the Capsid of Human Norovirus Is Highly Pressure Resistant. Appl. Environ. Microbiol..

[191] Lou F., DiCaprio E., Li X., Dai X., Ma Y., Hughes J., Chen H., Kingsley D.H., Li J. (2016). Variable High-Pressure-Processing Sensitivities for Genogroup II Human Noroviruses. Appl. Environ. Microbiol..

[192] Wilkinson N., Kurdziel A.S., Langton S., Needs E., Cook N. (2001). Resistance of Poliovirus to Inactivation by High Hydrostatic Pressures. Innov. Food Sci. Emerg. Technol..

[193] Govaris A., Pexara A. (2021). Inactivation of Foodborne Viruses by High-Pressure Processing (HPP). Foods.

[194] Ye M., Lingham T., Huang Y., Ozbay G., Ji L., Karwe M., Chen H. (2015). Effects of High-Hydrostatic Pressure on Inactivation of Human Norovirus and Physical and Sensory Characteristics of Oysters. J. Food Sci..

[195] Chen H., Hoover D.G., Kingsley D.H. (2005). Temperature and Treatment Time Influence High Hydrostatic Pressure Inactivation of Feline Calicivirus, a Norovirus Surrogate†. J. Food Prot..

[196] Kingsley D.H., Chen H. (2009). Influence of PH, Salt, and Temperature on Pressure Inactivation of Hepatitis A Virus. Int. J. Food Microbiol..

[197] Tang Q., Li D., Xu J., Wang J., Zhao Y., Li Z., Xue C. (2010). Mechanism of Inactivation of Murine Norovirus-1 by High Pressure Processing. Int. J. Food Microbiol..

[198] Rzeżutka A., Cook N. (2004). Survival of Human Enteric Viruses in the Environment and Food. FEMS Microbiol. Rev..

[199] Roos Y.H. (2020). Water and Pathogenic Viruses Inactivation—Food Engineering Perspectives. Food Eng. Rev..

[200] Grove S.F., Lee A., Lewis T., Stewart C.M., Chen H., Hoover D.G. (2006). Inactivation of Foodborne Viruses of Significance by High Pressure and Other Processes†. J. Food Prot..

[201] Choi M.-S., Jeon E.B., Kim J.Y., Choi E.H., Lim J.S., Choi J., Ha K.S., Kwon J.Y., Jeong S.H., Park S.Y. (2020). Virucidal Effects of Dielectric Barrier Discharge Plasma on Human Norovirus Infectivity in Fresh Oysters (Crassostrea Gigas). Foods.

[202] Boxman I., Tilburg J., Teloeke N., Vennema H., Jonker K., Deboer E., Koopmans M. (2006). Detection of Noroviruses in Shellfish in the Netherlands. Int. J. Food Microbiol..

[203] European Market Observatory for Fisheries and Aquaculture Products (EUMOFA), European Commission, Directorate General for Maritime Affairs and Fisheries (2019). The EU Fish Market: 2019 ed..

[204] European Market Observatory for Fisheries and Aquaculture Products (EUMOFA), European Commission, Directorate General for Maritime Affairs and Fisheries (2017). EU Consumer Habits Regarding Fishery and Aquaculture Products. Annex 1, Mapping and Analysis of Existing Studies on Consumer Habits of the European Union.

[205] Pouillot R., Smith M., Van Doren J.M., Catford A., Holtzman J., Calci K.R., Edwards R., Goblick G., Roberts C., Stobo J. (2021). Risk Assessment of Norovirus Illness from Consumption of Raw Oysters in the United States and in Canada. Risk Anal..

[206] Teunis P., Havelaar A., Vliegenthart J., Roessink G. (1997). Risk Assessment of Campylobacter Species in Shellfish: Identifying the Unknown. Water Sci. Technol..

[207] Strubbia S., Lyons B.P., Lee R.J. (2016). Geographical and Temporal Variation of E. Coli and Norovirus in Mussels. Mar. Pollut. Bull..

[208] Ilic N., Velebit B., Teodorovic V., Djordjevic V., Karabasil N., Vasilev D., Djuric S., Adzic B., Dimitrijevic M. (2017). Influence of Environmental Conditions on Norovirus Presence in Mussels Harvested in Montenegro. Food Environ. Virol..

[209] Teunis P.F.M., Le Guyader F.S., Liu P., Ollivier J., Moe C.L. (2020). Noroviruses Are Highly Infectious but There Is Strong Variation in Host Susceptibility and Virus Pathogenicity. Epidemics.

[210] Thebault A., Teunis P.F.M., Le Pendu J., Le Guyader F.S., Denis J.-B. (2013). Infectivity of GI and GII Noroviruses Established from Oyster Related Outbreaks. Epidemics.

[211] Atmar R.L., Opekun A.R., Gilger M.A., Estes M.K., Crawford S.E., Neill F.H., Ramani S., Hill H., Ferreira J., Graham D.Y. (2014). Determination of the 50% Human Infectious Dose for Norwalk Virus. J. Infect. Dis..

[212] Kirby A.E., Teunis P.F., Moe C.L. (2015). Two Human Challenge Studies Confirm High Infectivity of Norwalk Virus. J. Infect Dis..

[213] Van Abel N., Schoen M.E., Kissel J.C., Meschke J.S. (2017). Comparison of Risk Predicted by Multiple Norovirus Dose-Response Models and Implications for Quantitative Microbial Risk Assessment: Comparison of Risk Predicted by Multiple Norovirus Dose-Response Models. Risk Anal..

[214] Nordgren J., Svensson L. (2019). Genetic Susceptibility to Human Norovirus Infection: An Update. Viruses.

[215] Lowther J.A., Avant J.M., Gizynski K., Rangdale R.E., Lees D.N. (2010). Comparison between Quantitative Real-Time Reverse Transcription PCR Results for Norovirus in Oysters and Self-Reported Gastroenteric Illness in Restaurant Customers. J. Food Prot..

[216] Toffan A., Brutti A., De Pasquale A., Cappellozza E., Pascoli F., Cigarini M., Di Rocco M., Terregino C., Arcangeli G. (2014). The Effectiveness of Domestic Cook on Inactivation of Murine Norovirus in Experimentally Infected Manila Clams (*Ruditapes Philippinarum*). J. Appl. Microbiol..

[217] Biswas D., Micallef S.A. (2019). Safety and Practice for Organic Food.

[218] Hewitt J., Rivera-Aban M., Greening G.E. (2009). Evaluation of Murine Norovirus as a Surrogate for Human Norovirus and Hepatitis A Virus in Heat Inactivation Studies. J. Appl. Microbiol..

